# Anemia and Its Effect on Cardiovascular Findings in Obese Adolescents

**DOI:** 10.4274/tjh.2018.0103

**Published:** 2018-08-05

**Authors:** Öner Yıldırım, Tülay Demircan, Özlem Tüfekçi, Özgür Kızılca, Pınar Kuyum, Mustafa Kır, Ayhan Abacı, Nurettin Ünal, Nur Arslan, Ece Böber, Şebnem Yılmaz, Hale Ören

**Affiliations:** 1 Dokuz Eylül University Faculty of Medicine, Department of Pediatrics, İzmir, Turkey; 2 Dokuz Eylül University Faculty of Medicine, Department of Pediatric Cardiology, İzmir, Turkey; 3 Dokuz Eylül University Faculty of Medicine, Department of Pediatric Hematology, İzmir, Turkey; 4 Dokuz Eylül University Faculty of Medicine, Department of Pediatric Gastroenterology and Metabolism, İzmir, Turkey; 5 Dokuz Eylül University Faculty of Medicine, Department of Pediatric Endocrinology, İzmir, Turkey

**Keywords:** Anemia, Cardiac function, Inflammation, Iron deficiency, Obesity

## Abstract

**Objective:**

We assessed the effect of anemia on cardiovascular findings in obese adolescents.

**Materials and Methods:**

We studied 29 anemic and 33 nonanemic obese adolescents, and 33 nonobese healthy adolescents. These three groups were investigated for clinical and laboratory features of anemia and obesity. Echocardiography was used to examine cardiac functions.

**Results:**

The anemia was mild (mean hemoglobin: 11.67±0.79 g/dL), ferritin level was significantly low, and C-reactive protein and fibrinogen levels were significantly high in anemic obese patients. Increased cardiac pulse and echocardiographic findings, which may be indicative of early left ventricular diastolic dysfunction, were present in these patients.

**Conclusion:**

Anemia may develop due to iron deficiency and chronic inflammation in obese adolescents. Even mild anemia may cause increased heart rate and affect left ventricular diastolic functions. Diet programs for obese children should be carefully planned to avoid iron deficiency anemia, which may worsen the cardiac events in long-term follow-up.

## Abstract (tr)

**Amaç:**

Obez adölesanlarda aneminin kardiyovasküler bulgular üzerine etkisinin araştırılması amaçlanmıştır.

**Gereç ve Yöntemler:**

Çalışmaya 29 anemik, 33 anemik olmayan obez adölesan ve 33 obez olmayan sağlıklı adölesan dahil edildi. Bu üç grup, anemi ve obesitenin klinik ve laboratuvar bulguları açısından değerlendirildi. Kardiyak fonksiyonları değerlendirmek için ekokardiyografi kullanıldı.

**Bulgular:**

Anemik obez hastalarda anemi hafifti (ortalama hemoglobin 11,67±0,79 g/dL), ferritin seviyesi anlamlı olarak düşük, C-reaktif protein ve fibrinojen düzeyleri anlamlı olarak yüksek bulundu. Bu grupta kardiyak nabız anemik olmayan obez adölesanlarınkine göre anlamlı yüksekti ve ekokardiyografik incelemede anemik obez grupta erken ventriküler diyastolik disfonksiyon göstergesi olabilecek bulgular saptandı.

**Sonuç:**

Obez adölesanlarda demir eksikliği ve kronik enflamasyona bağlı anemi gelişebilir. Hafif anemi varlığı bile kardiyak nabızda artışa ve sol ventrikül diyastolik fonksiyonlarında etkilenmeye neden olabilir. Bu nedenle obez çocuklarda uzun dönemde kalp fonksiyonlarının olumsuz yönde etkilenmemesi açısından diyet programları demir eksikliği anemisini önleyecek şekilde dikkatlice planlanmalıdır.

## Introduction

The prevalence of childhood obesity has progressively increased in the world in the last decades due to sedentary life style and poor dietary habits [1,2]. Childhood obesity is a major risk factor for development of cardiovascular diseases in adulthood [3,4,5,6]. On the other hand, anemia is another well-defined risk factor that has a negative impact on the prognosis of cardiovascular diseases [7,8,9]. The cardiac problems in anemic obese adolescents are not well known. The purpose of this study was to assess the effect of anemia on cardiovascular findings in obese adolescents by means of standard, pulsed-wave Doppler (PWD), and tissue Doppler imaging (TDI) echocardiography.

## Materials and Methods

Adolescent patients admitted to our hospital with exogenous obesity between the ages of 12 to 18 years were included. The study group was divided into two groups as anemic obese (n=29) and nonanemic obese (n=33) patients. Those who had endogenous obesity, infection, chronic use of medications, or other accompanying diseases were excluded. Healthy adolescents (n=33) whose body mass indexes (BMIs) were between the 3^rd^ and 85^th^ percentiles were included as the control group. 

Obesity was defined as a BMI at or above the 95^th^ percentile for children and teenagers of the same age and sex. BMI is calculated by dividing a person’s weight in kilograms by the square of height in meters [10]. Anemia was defined according to the World Health Organization as hemoglobin value of ≤12 g/dL in women and ≤13 g/dL in men [11]. Hypertension was defined by a systolic and/or diastolic blood pressure at or above the 95^th^ percentile for children and teenagers of the same age and sex [12]. 

Clinical data and results of laboratory measurements of patients were obtained from the hospital records, including complete blood cell count; renal, liver, and thyroid function tests; serum glucose, insulin, insulin resistance, lipid, fibrinogen, and C-reactive protein (CRP) levels; and iron parameters. 

Echocardiography was performed after 15 min of resting by a pediatric cardiologist. Standardized M-mode echocardiography, PWD, and TDI echocardiography were performed to evaluate the status and functions of the heart [13]. By using M-mode echocardiography, interventricular septum diastolic diameter (IVSDD), left ventricular end-diastolic diameter, left ventricular posterior wall diastolic diameter (LVPWDD), left ventricular end-systolic diameter, ejection fraction (EF), left ventricular mass (LVM), and LVM index (LVMI) were calculated. Early diastolic mitral flow (E-wave), late diastolic mitral flow (A-wave), and early mitral to late mitral flow ratio (E/A) were found using PWD. Systolic myocardial velocity (S), late diastolic myocardial velocity (Em), early diastolic myocardial velocity (Am), ratio of early to late diastolic myocardial velocity (Em/Am), isovolumetric relaxation time, and myocardial performance index were calculated using TDI echocardiography.

All statistical analyses were performed using SPSS 15 (SPSS Inc., Chicago, IL, USA). Differences between groups for categorical variables were compared by chi-square test. The Student t-test and Mann-Whitney U test were used for the comparison of continuous variables. One-way analysis of variance (ANOVA) and Kruskal-Wallis tests were used for the comparison of more than two groups.

## Results

Demographic data and clinical features of the groups are given in Table 1. The values of hemoglobin, mean corpuscular volume, red cell distribution width, iron parameters, fibrinogen, and CRP are shown in Table 2. Test results including serum glucose, insulin, insulin resistance, lipid profile, and renal, liver, and thyroid function tests did not differ among the three groups (p>0.05). 

M-mode and TDI echocardiographic parameters are given in Table 3 and Table 4. As seen in Table 3, there were significant changes of LVM, LVMI, LVPWDD, and IVSDD in obese patients. PWD measurements demonstrated that the E-wave and A-wave showed significant differences between the three groups (p=0.012, p=0.013) and between anemic and nonanemic obese groups (p=0.016, p=0.039). The E/A ratio was not statistically significant between the three groups (p=0.751). 

## Discussion

In our study, a significant proportion of anemic obese children were found to be on a diet to lose weight. Their ferritin levels were significantly lower even though there were signs of chronic inflammation, such as high levels of fibrinogen and CRP. Anemia may be seen in the obese population due to poor dietary habits and as a result of chronic inflammatory condition [14,15,16,17,18]. In obese patients, adipose tissue secretes proinflammatory cytokines that restrict erythropoiesis [17,18]. On the other hand, obesity-associated inflammation is closely linked to iron deficiency and involves impaired duodenal iron absorption associated with low expression of duodenal ferroportin and elevated hepcidin levels [14,19]. Iron deficiency and anemia may change mitochondrial and cellular energy homeostasis and increase the inactivity and fatigue of obese patients [19]. 

Anemia may cause hemodynamic changes, cardiomegaly, and left ventricular hypertrophy in the long-term period [9,20]. EF is one of the most commonly used parameters in evaluation of left ventricular systolic function. EF was not impaired in our study. Studies demonstrated that EF does not decrease in the early period of obesity [21,22,23]. It has been reported that these changes correlate with the degree and duration of anemia [9,20,24]. In a recent study, Zhou et al. [24] demonstrated that LV remodeling and LV systolic dysfunction occurred in patients with iron deficiency anemia when the hemoglobin level was in the range of 6-9 g/dL. In our study, the mean hemoglobin value was 11.67±0.79 g/dL; it can be concluded that mild anemia in the obese population does not deteriorate systolic dysfunction. 

Tachycardia, a well-known complication of anemia, develops as a compensatory response of the heart to inadequate tissue oxygenation caused by decreased erythroid mass [9]. In our study, the anemic obese group was found to have significantly higher cardiac pulse rates than the nonanemic obese group, even though the anemia was mild. The changes in E- and A-waves seen in PWD might be caused by increased heart rates in our anemic obese group, which may be indicative of early subclinical ventricular diastolic dysfunction [21,23,25,26]. 

Regarding the cardiac geometry, an increased LVMI has been shown in obese children [6]. Sharpe et al. [27] demonstrated that BMI is directly related to LVMI. An increased LVMI results in ventricular hypertrophy, which eventually results in left ventricular diastolic dysfunction [23,25,26,27,28,29,30]. Similarly, in our study, measurements of LVM, LVMI, LVPWDD, and IVSDD were found to be increased in both obese groups compared to the healthy control group. 

In this study, the number of patients was relatively low and the anemia was mild, so we recommend further studies with larger samples of obese adolescents with different stages of anemia for more accurate investigation of effects of anemia in obese adolescents. Follow-up of these adolescents is also important to provide a prompt therapeutic approach and better outcome.   

## Conclusion

Anemia may develop due to iron deficiency and chronic inflammation in obese adolescents. Our study suggests that blood pressure, heart rate monitoring, and echocardiographic measurements should be carefully checked in anemic obese adolescents at frequent intervals for early detection of hypertension, tachycardia, and left ventricular diastolic dysfunction. Even mild anemia may cause increased heart rate and change the left ventricular diastolic functions in obese adolescents. Diet programs of obese children should therefore be carefully planned to avoid iron deficiency anemia, which may worsen the cardiac outcome in long term follow-up.

## Figures and Tables

**Table 1:**
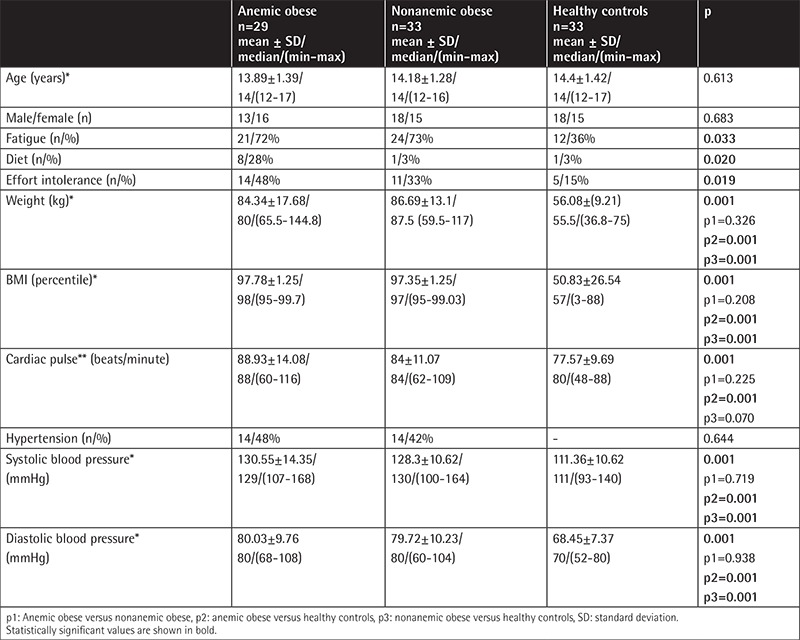
Demographic and clinical features of the three groups.

**Table 2:**
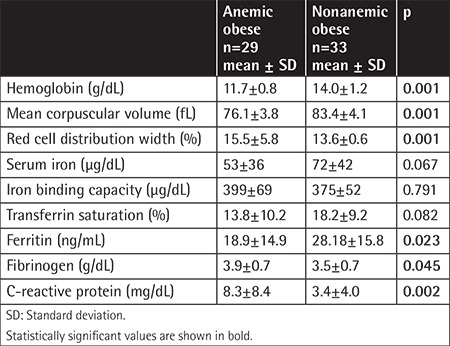
Hemoglobin, erythrocyte indexes, iron parameters, fibrinogen, and C-reactive protein levels of anemic and nonanemic obese adolescents.

**Table 3:**
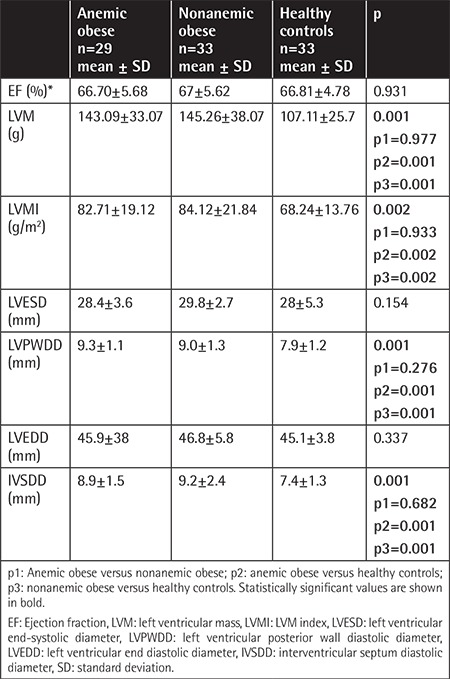
Comparison of M-mode echocardiographic parameters between the three groups.

**Table 4:**
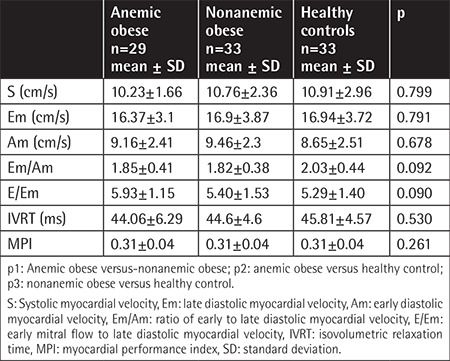
Comparison of the tissue Doppler image parameters between the three groups.
